# Crossroads at the Origin of Prebiotic Chemical Complexity: Hydrogen Cyanide Product Diversification

**DOI:** 10.1021/acs.jpca.3c01504

**Published:** 2023-05-11

**Authors:** Hilda Sandström, Martin Rahm

**Affiliations:** Department of Chemistry and Chemical Engineering, Chalmers University of Technology, Gothenburg SE-412 96, Sweden

## Abstract

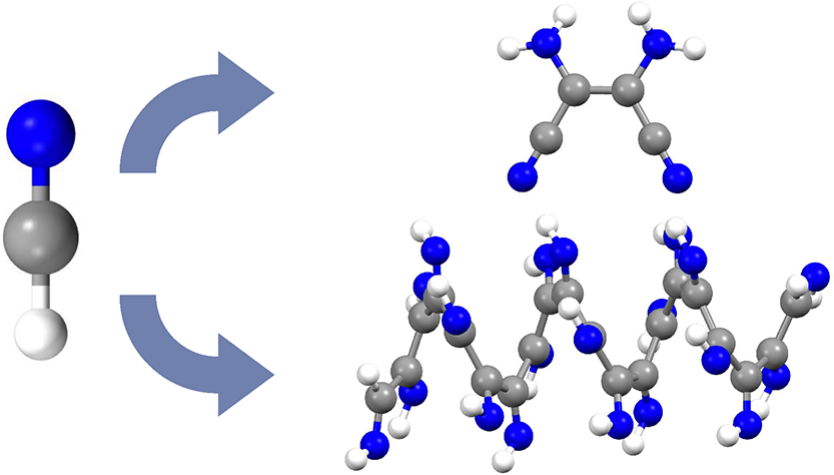

Products of hydrogen cyanide (HCN) reactivity are suspected to play important roles in astrochemistry and, possibly, the origin of life. The composition, chemical structure, and mechanistic details for formation of products from HCN’s self-reactions have, however, proven elusive for decades. Here, we elucidate base-catalyzed reaction mechanisms for the formation of diaminomaleonitrile and polyimine in liquid HCN using ab initio molecular dynamics simulations. Both materials are proposed as key intermediates for driving further chemical evolution. The formation of these materials is predicted to proceed at similar rates, thereby offering an explanation of how HCN’s self-reactions can diversify quickly under kinetic control. Knowledge of these reaction routes provides a basis for rationalizing subsequent reactivity in astrochemical environments such as on Saturn’s moon Titan, in the subsurface of comets, in exoplanet atmospheres, and on the early Earth.

## Introduction

In this work, we evaluate reaction mechanisms hypothesized for the polymerization of hydrogen cyanide (HCN) using ab initio molecular dynamics simulations. HCN is ubiquitous in the solar system and beyond, having been detected on comets,^1^ various planets,^2,3^ dwarf planets,^4^ in the interstellar medium,^5^ and in the atmosphere of Saturn’s moon Titan.^6^ HCN is also suspected to have formed in the atmosphere of the early Earth, as a product of photochemical reactions between nitrogen and methane^7^ or following superflares, shockwaves, or discharge chemistry.^8,9^ Given the prevalence of HCN, its reactions likely play a role in the chemical evolution of planets, including their habitability.^10,11^ Understanding the reactivity of HCN is, consequently, important to astrochemistry, prebiotic chemistry, and, through them, astrobiology.

HCN lies at the roots of reaction networks proposed to explain the origins of RNA, protein, and lipid precursors.^12,13^ Biomolecular building blocks, such as nucleobases, cofactors, and amino acids,^14−17^ have been detected among the reaction products of HCN polymerization experiments. Several HCN-derived polymers are also functional materials with a wide range of properties, making them suitable as catalysts and coatings.^18,19^

**Figure 1:**
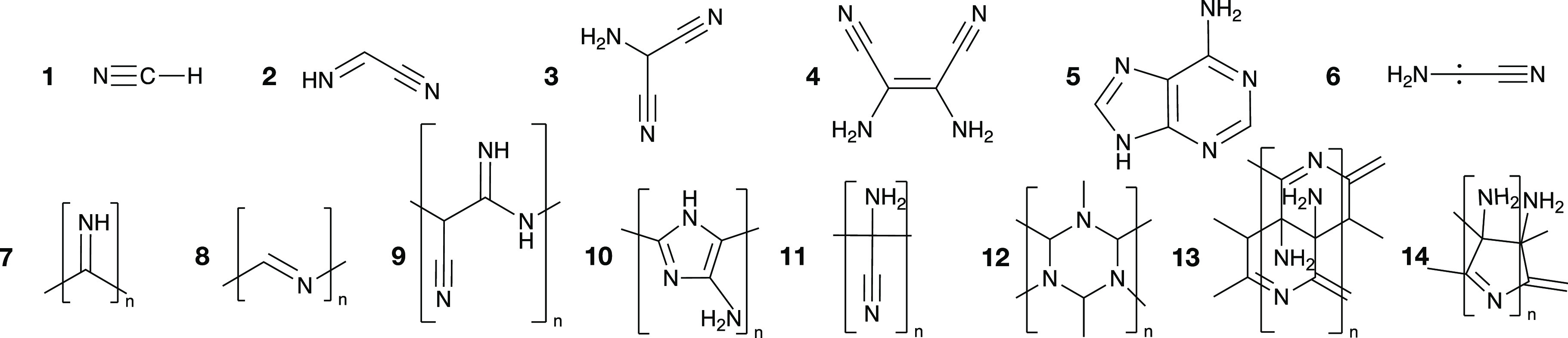
HCN (**1**) and some of the potential products of its self-reaction chemistry. Top: selection of molecules that may constitute reaction intermediates and that have either been detected or proposed to exist in HCN reaction mixtures: iminoacetonitrile (**2**),^21^ aminomalononitrile (**3**),^14,35^ diaminomaleonitrile (DAMN, **4**),^34^ adenine (**5**),^14^ and aminocyanocarbene (**6**).^28,33,36^ Bottom: a selection of proposed polymer backbones: polyimine (**7**),^26^ nitrogen-substituted polyacetylene (**8**),^28,33^ polyaminomalononitrile (**9**),^31^ polyaminoimidazole (**10**),^37^ polyaminocyanomethylene (**11**),^21^ extended triazine networks (**12**),^28,38^ Völker’s “double-ladder” model (**13**),^21,29^ and Umemoto’s “single-ladder” model (**14**).^29^

Experimental attempts at elucidating the structure of products resulting from the self-reaction of HCN (into polymers and molecules) have proven challenging. The difficulty arises in part because the often-complex products are insoluble in most solvents, which hinders separation and characterization.^20^ Analyses of HCN reaction products (including those formed in aqueous solution) have found evidence for a wide variety of functional groups, including amines,^21−23^ amides,^22,24^ imines,^25,26^ carbodiimides, triazines, nitriles, and carbonyls^27^ among others. A number of different polymer backbones have also been proposed to explain these observations, and different polymerization pathways are under active study (Figure 1).^21,23,25,26,28,29^

Outstanding questions in the study of HCN reactions include not only the nature of products but also their precursors, i.e., which molecular intermediates may give rise to the final products. The top of Figure 1 shows several such possible intermediates, along with suggested polymer structures. Völker has proposed one possible scenario: that the HCN dimer iminoacetonitrile **2** reacts to form **11**,^21^ a polymer which, in turn, might be an intermediate for ring-containing structures such as **13** and **14**.^21,29^ Matthews and Moser have suggested that aminomalononitrile **3** forms polymer **9** via the high-energy carbene **6**.^30^ Compounds such as **9** might, hypothetically, convert into polypeptides upon hydrolysis.^19,31,32^ Mamajanov and Herzfeld and Mozhaev et al. have proposed that **6** might function as an initiator for the formation of **8**.^28,33^ He et al. have proposed a base-catalyzed pathway to form polyimine, **7**, directly from HCN.^26^ The details of these suggestions are mostly speculative, and the invoked intermediates **2**, **3**, and **6** have so far escaped experimental detection. In fact, the only proposed polymer precursor, besides HCN, whose presence is well-established in reaction mixtures, is the tetramer diaminomaleonitrile, **4**,^25,34,35^ commonly abbreviated as DAMN.

Interest in the role of **4** in HCN polymerization originates with the work by Ferris and colleagues, who argued that **4** and not HCN (nor its dimer or trimer) is the direct precursor to observed reaction products.^39^ For example, **4** has been identified in HCN polymerization experiments using nuclear magnetic resonance (NMR) spectroscopy,^28^ chromatography,^35^ and by the precipitation of its crystal from HCN reaction mixtures.^23^ The established formation of **4** has garnered the molecule a central role in many polymerization and HCN reaction mechanism hypotheses.^19^ After the suggestions by Ferris, **4** has been proposed as an intermediate to most (**9**, **10**, **11**, **13**, and **14**) of the polymers shown in Figure 1.^21,29,37,40^ Importantly, **4** is also hypothesized to be an intermediate for the formation of the nucleobase adenine, **5**, in turn central to theories of prebiotic HCN-based chemistry (refs (35, 16) and references therein). Despite extensive evidence for its presence in reaction mixtures, we stress that **4** has not been experimentally proven to be a *necessary* intermediate in HCN’s transformation into larger molecules or polymers.

The results of several experimental studies suggest that **4** is too unreactive to be the basic building block of polymers formed in conventional HCN polymerization experiments. For example, Ruiz-Bermejo and colleagues have shown that base-catalyzed polymerization of **4** is very ineffective close to or below room temperature in aqueous solution [small yields of polymers (∼1 wt %) were only observed in the presence of ammonia^25^]. Efficient polymerization of **4** has been reported above 398 K in the neat solid,^37,41,42^ above 449 K in a neat melt,^41^ and above 353 K in aqueous solution at pH 9.2.^25,40^ In all cases, efficient polymerization of **4** requires considerably higher temperatures compared to conditions in typical HCN polymerization/reaction experiments. A study by He et al. reported that only 10% of the polymer product could be attributed to those suggested to form from **4**.^26^ Meanwhile, Mamajanov and Herzfeld have proposed that **4** is an unreactive side product during HCN polymerization.^28^ In other words, the role of **4** might, in principle, be several: it may be a largely unreactive side product; act as an intermediate in HCN polymerization; or, as Sanchez et al. have suggested, **4** might catalyze HCN polymerization.^35^

Interest in **7** stems in part from predictions of extensive polymorphism and a broad absorption spectrum in the visible region.^18^ The photochemical properties of this polymer combined with its ability to form strong directional hydrogen bonds are also suggestive of a potential for catalysis. These properties, and a predicted weakly exothermic formation of **7**, may, in principle, facilitate dynamic chemistry in cold astrochemical environments, such as those on Titan.^18^ Experimental evidence supporting the formation of **7** (up to 75 wt %) from HCN relies on the detections of imine groups in multidimensional NMR studies.^25,26^

The unique electronic structure and relatively high energy of **7** compared to many other polymers likely make the material prone to further reactions. Following this line of thought, Ruiz-Bermejo and co-workers have proposed that **7** can provide a possible explanation for heterocyclic motifs observed in HCN polymerization experiments.^22^ We will not resolve the formation of heterocycles from HCN in this work but focus on comparing plausible formation mechanisms of both **4** and **7** under identical, and realistic, reaction conditions: base-catalyzed reaction of liquid HCN.

### Pathways toward DAMN and Polyimine

**Figure 2:**
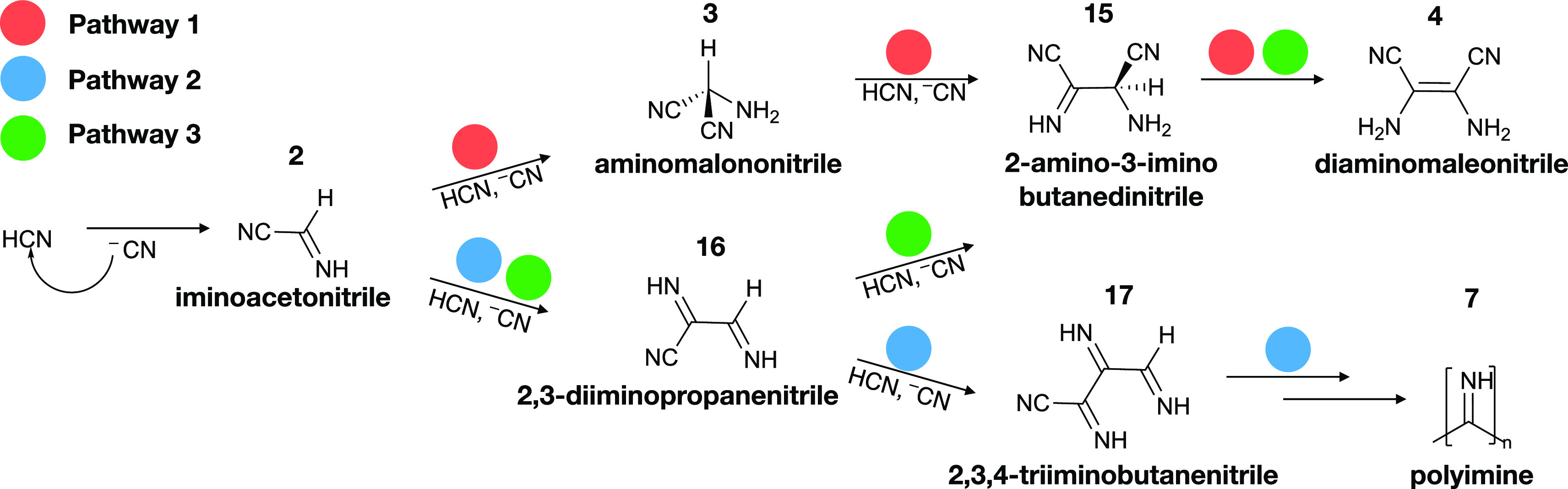
Proposed formation pathways to diaminomaleonitrile (**4**) and polyimine (**7**) represent possible beginnings of chemical diversification from simple origins. Pathway 1 (red) involves two nucleophilic additions of cyanide anions, first onto an sp^2^ carbon of iminoacetonitrile (**2**) and secondly onto the nitrile group of the trimer aminomalononitrile (**3**). The additions are accompanied by proton transfers. The formed intermediate 2-amino-3-imino butanedinitrile (**15**) can form **4** via imine–enamine tautomerization. In pathway 2 (blue), **7** forms through successive additions of cyanide anions onto the nitrile group of a growing polymer chain. Pathway 3 (green) is an alternative formation route of **4**. The first step in pathway 3 is the same as in pathway 2 where 2,3-diiminopropanenitrile (**16**) forms from **2**. In the second step of pathway 3, **15** forms through an addition of a cyanide anion onto **16**.

Figure 2 outlines three contesting base-catalyzed reaction routes to the formation of **4** and **7**. Pathway 1 was proposed by Ferris and coworkers to explain the formation of **4**.^34,35^ In this mechanism, **4** forms following successive nucleophilic additions of cyanide anions coupled to proton transfers. The first intermediate in this sequence of reaction steps is **2**, the most stable HCN dimer.^43^ We have previously detailed the kinetics and thermodynamics of the formation of **2** using ab initio molecular dynamics simulations and found it feasible near ambient conditions.^44^ The second step in pathway 1 is the formation of **3** through addition of a cyanide anion to the sp^2^ carbon of **2**. This reaction step was also included by Oró in his suggested mechanism for adenine formation.^14^ Next, in pathway 1, **3** reacts with another cyanide anion to form 2-amino-3-imino butanedinitrile, **15**, a tautomer of **4**. In the final step, **15** rearranges into **4** by imine–enamine tautomerization. Initial evidence in favor of pathway 1 was presented by Ferris and colleagues, who reported the production of **4** following addition of **3** to a cyanide solution.^35^

Despite extensive subsequent studies (refs (34, 19) and references therein), neither the dimer nor trimer intermediates have been experimentally detected, which in turn implies that the equilibrium is strongly shifted toward **4**.

Pathway 2 was proposed by He et al. and outlines a competing base-catalyzed mechanism in which **7** forms instead of **4**.^26^ In this alternative, polymer growth proceeds through successive additions of cyanide anions onto terminal nitrile groups. Pathway 3, also proposed by He et al., is an alternative in which the HCN trimer 2,3-diiminopropanenitrile, **16,** serves as a precursor to **4**.^26^

Understanding which of these three mechanisms are active is an essential step toward untangling many hypotheses regarding HCN’s self-reaction chemistry and in extension the role of HCN in prebiotic chemistry. Unfortunately, discerning the overall selectivity of these reactions is extremely challenging experimentally. The formation of **4** and **7** shares a set of similarities: both reactions reportedly proceed at similar temperatures, and both are base-catalyzed. The proposed pathways to **4** and **7** also share a number of early intermediates (**2** and **16**). Early theoretical work by Loew et al. has indicated that the two carbon atoms of **2** are equally electrophilic, suggesting a similar reactivity.^45^ Finally, as both **4** and **7** are suspected intermediates of more complex reaction products, it is unlikely that comparing their respective experimental yields will be productive. Quantum chemical simulations allow the testing of these competing hypotheses in well-defined conditions. Our methodology relies on mapping the free energy profile of the reaction pathways with steered ab initio molecular dynamics simulations (see the Methods section). To this end, we construct a free energy profile through umbrella sampling, a method whereby the reacting system is simulated at different points along a reaction coordinate using an applied bias potential. Ab initio molecular dynamics simulations can describe strong intra- and intermolecular interactions as well as reactive and dynamic behavior of solvents such as HCN and water that are typically used in HCN polymerization experiments. Herein, our solvent model is that of pure liquid HCN at 278 K (matching conditions described in ref (37). It is essential to properly consider solvent effects for reactions in HCN because (1) similar to water, liquid HCN has the possibility to both accept and donate hydrogen bonds and build a strong hydrogen bond network. (2) HCN has the sixth highest dielectric constant of any liquid.^46,47^ At 278 K, the dielectric constant is 144.8, 1.7 times larger than that of water at the same temperature.^46,48^ (3) Solvent effects are additionally larger in reactions involving ions, which we study here.

## Methods

### Conformational Transition State Search

Molecular models were used first to identify transition states (TS) of all reactions under study (Figure S3). Geometry optimizations were performed in Gaussian 16, revision B.01,^49,50^ using the Perdew–Burke–Ernzerhof (PBE) functional^51^ and Grimme’s D3 dispersion correction.^52^ Wavefunctions were described with a 6-31G(d,p) basis set because of its similarity to the DZVP-Goedecker–Teter–Hutter (GTH) basis set^53^ used in subsequent molecular dynamics simulations. The effect of HCN solvation was in this first step modeled using a polarizable continuum model^50^ relying on the default settings for water, while adjusting the dielectric constant to that of HCN at 278 K (ε = 144.8).^46^ Subsequent calculations with B3LYP-D3^54^ and M06-2X^55^//B3LYP-D3 levels of theory were used to validate the identity of lowest-energy conformers (see Figures S1–S3). Results using M06-2X and B3LYP-D3 differ by less than 1 kcal/mol in all relevant cases. TS structures identified as lowest in energy were subsequently solvated and equilibrated in liquid HCN using molecular dynamics simulations (see below and the Supplementary Information for details).

### Molecular Simulations

All simulations were performed with CP2K v6.1^56^ and included a cubic box of liquid HCN and a cyanide anion subjected to periodic boundary conditions. The simulation box was singly negatively charged. The length of the simulation box was 15.94 Å, set to reproduce the experimental density of liquid HCN at 278 K (0.709 g/cm^3^).^57^ The number of HCN solvent molecules was chosen such that the sum of HCN units in the oligomer and in the solvent (counting the cyanide anion) equaled 64. This choice corresponds to a CN^–^ (base) concentration of ca. 0.4 M and is similar to past experimental work. For example, Mamajanov and Herzfeld^28^ and He et al.^26^ used 0.5 wt % of triethylamine and ammonia, respectively. Assuming complete protonation of these strong bases, these conditions correspond to CN^–^ concentrations of 0.03 and 0.21 M, respectively. For similar experiments in aqueous solution (see e.g., ref (25)), a strong base is typically added to adjust the pH to the pKa of HCN, in which case there is an equal amount of CN^−^ and HCN. In other words, for a representative 1 M HCN solution kept near pH = 9.2, the concentration of CN^−^ and HCN are both ∼0.5 M.

Our simulations were carried out in an NVT ensemble at 278 K and relied on the DZVP-GTH basis set^53^ and GTH pseudopotentials with an energy cutoff of 280 Rydberg. The canonical sampling through a velocity rescaling thermostat^58^ was used with a time constant of 50 fs for production runs and 1 fs for equilibrations except for the reference simulations of **2** (for details, see “Construction of path collective variables for steered simulations” in the Supplementary Information).

### Reference State Simulations

Reference coordination patterns of both reactants and products were obtained from 20 ps simulations that followed 5 ps equilibration runs. Path collective variables were then obtained, as outlined in the Supplementary Information and refs (59, 60). The path collective variable *s* defines the reaction coordinate.

### Umbrella Sampling and Committor Analysis

Umbrella sampling simulations were made to evaluate the free energy barrier in solution. Each TS was equilibrated for 20–25 ps while constrained to its *s* coordinate. A set of trajectories to reactants and products were then generated from the equilibrated transition states. Structures spaced at most 0.033 *s* units (∼4%) apart were selected from the trajectories and used as starting points for umbrella sampling simulations. Force constants for the simulation windows (available in the Supplementary Information) were chosen to not exceed , where σ^2^ is the variation of the *s* coordinate in a typical simulation, *k*_b_ is the Boltzmann constant, and *T* is the temperature. The umbrella sampling simulations were run for 10 ps after a 1 ps equilibration. The PLUMED 2.5 library^61^ was used to steer the simulations. The weighted histogram analysis method^62^ version 2.0.9 was used to construct energy profiles and compute their standard deviations through block-averaging (Figure S6). Positions of the transition states in an explicit solvent were determined as the highest points on the free energy curves shown in Figure S7.

In this study, we approximate Helmholtz free energy of reaction as Gibbs free energy of reaction because we expect the *PV* term to vary little compared to the internal energy. This approximation is common in dynamics studies of condensed phase systems where the associated molar volumes are small.

## Results and Discussion

**Figure 3:**
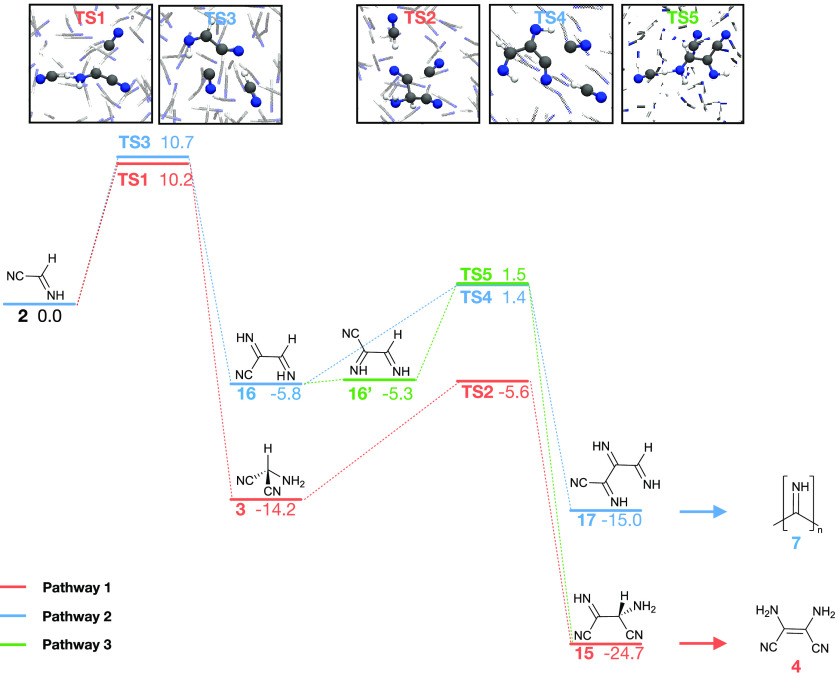
Free energy profile of the first reaction steps leading to diaminomaleonitrile (**4**) and polyimine (**7**). Bottom: computed relative Gibbs free energy (kcal/mol) of states leading to the formation of **4** and **7** in liquid HCN at 278 K. Compound 2,3,4-triiminobutanenitrile (**17**) is a precursor to **7**, and amino-3-imino butanedinitrile (**15**) is a precursor to **4**. **16**′ and **16** correspond to two isomers of diiminopropanenitrile that are 0.5 kcal/mol apart (see the Supplementary Information, Figure S1). Top: representations of transition states taken from snapshots of our simulations.

Figure 3 shows the computed Gibbs free energy profiles of the three considered pathways alongside representative snapshots that correspond to transition states. The quantum mechanical level of theory (PBE-D3) used to generate these data is a compromise between computational feasibility and accuracy. Our method is known to underestimate association and proton transfer reaction barriers by 3.4 and 4.0 kcal/mol (mean absolute deviations), respectively.^63,64^ For example, the barrier for the formation of **2** is underestimated by ∼5 kcal/mol at the PBE-D3 level of theory compared to the more accurate B3LYP-D3 method.^44^ At the same time, PBE is known to estimate the cooperativity of hydrogen bonds in HCN clusters to within 1 kcal/mol of CCSD(T) results.^65^ PBE-D3 is also often able to correctly identify the lowest energy conformer of organic materials more generally.^66^ We stress that our goal here is not to determine absolute barrier heights but rather to compare barriers of competing pathways. Nevertheless, we have validated our choices of low-lying conformers against both B3LYP-D3 and M06-2X calculations (see Figures S1–S3).

Our investigation focuses on comparing the steps following the formation of the HCN dimer **2**, the proposed first shared intermediate of **4** and **7**. Our best estimate of the barrier for formation of **2** is 21.8 kcal/mol.^44^ This barrier is in good agreement with experimental estimates at 283–313 K by Sanchez et al., who studied formation of **4**.^35^ As we will show, our modeling of the subsequent reaction steps supports a premise in which formation of **2** is the rate-determining step for formation of both **4***and***7** (and likely other products as well). Our simulations predict exceedingly similar barrier heights in subsequent competing reaction steps: computed barriers for the second steps in pathways 1 and 2 differ by only 0.5 kcal/mol. The corresponding kinetics predicted for the third step in each pathway are similarly comparable: the two lowest barriers leading to **15** and **17** through **TS5** and **TS4**, respectively, are calculated to be only 0.1 kcal/mol apart. The difference between the lowest barriers in each step is within the statistical uncertainty of our free energy profiles (see the Supplementary Information, Figure S7). The statistical uncertainties arise due to limited sampling in the simulations and lie in a range of 1.1–2.9 kcal/mol (standard deviation, see the Methods and Supplementary Information, Figure S8). In other words, whereas we predict equal kinetics for the two competing pathways, their rates may be up to 3 orders of magnitude different at 278 K (this estimate was made using the Eyring equation). We note that barriers for the second and third steps in all pathways are 5–10 kcal/mol *smaller* than the preceding formation of **2**, rendering the first process rate-determining. The barrier height of the third step (**16** → **7**) in pathways 2 and 3 is representative also for continued growth of **7** (see the Supplementary Information, Figure S4). Quantum mechanical tunneling may be expected to play a role in reaction steps involving hydrogen transfer. We have not considered such effects in our simulations due to the associated computational cost. One prime example is the final **15** → **4** tautomerization, which we have not studied but expect to be comparatively fast.^67^ Consequently, we predict little or no kinetic preference for either of the reaction mechanisms leading to **7** and **4**. We note that solvent participation is important in all transition states, **TS1–5**, both through explicit coordination and proton donation by HCN molecules. Moreover, in analogy to keto-enol tautomerization in polar liquids,^68^ we expect solvent mediation to be important for the **15** → **4** tautomerization in HCN liquid.

All products are clearly thermodynamically downhill, with an ∼10 kcal/mol preference for **15** over **17**. This difference grows to 24 kcal/mol between **4** and **17** following **15** → **4** tautomerization (Figure S2). The average change in Gibbs free energy per reaction step toward **7** is ∼ −7.5 kcal/mol, close but marginally more exergonic compared to previous estimations for the polymerization of HCN into **7** in the solid state (< −5.6 kcal/mol).^18^ This difference in predicted thermodynamics suggests that the initial steps of **7** formation are slightly more favored compared to bulk polymerization into **7**. Alternatively, it means that the reaction to **17** (and onward to **7**) is more favored in the liquid state.

The predicted kinetics and thermodynamics for the formation of **4** and **7** have several implications for the interpretation of experimental results. Whereas **4** is clearly the thermodynamically favored product, one should not rule out structures reminiscent of **7** playing a major role in HCN polymerization experiments. Oligomers of polyimine (**7**) will be of relatively high energy, and their feasible formation suggests that they may play important roles as reactive intermediates leading to larger structures. For example, there likely exist substantial thermodynamic driving forces for converting oligomers of **7** into heterocyclic motifs, as suggested by Ruiz-Bermejo et al.^25^

### Consequences for Astrochemistry in Different Environments

We have previously predicted^44^ that the formation of **2** is feasible in periodically heated comets and under certain conditions on the early Earth but exceedingly slow in many colder astrochemical environments where concentrated HCN may be found. Of note is that we here predict all subsequent reaction steps to be considerably *faster* than the initial formation of **2**. Our best estimate for the largest barrier height following the formation of **2** is ∼16 kcal/mol (obtained by correcting for the ∼5 kcal/mol underestimation of barrier heights by our method^63,64^). In other words, once HCN polymerization, e.g., to **7**, is initiated, it may proceed even in rather frigid conditions. If the first step can be circumvented, and we assume a barrier to continued polymerization of 16 kcal/mol, then such processes can proceed at temperatures as low as ∼150 K. The latter estimate was reached using the Eyring equation, assuming first-order reaction kinetics and a reaction time-scale of 1000 years.

What compounds might be realistic initiators for HCN polymerization, and in which conditions would they be relevant? We speculate that some other nitriles and ions present, e.g., in the atmosphere of Titan, as well as radiation (UV/vis or ionizing) can supply different forms of initiation, circumventing the initial formation of **2**. Cyanohydrins, such as glyconitrile, are one example that has been shown to accelerate aqueous HCN polymerization.^69^ The calculated activation barriers for cyanide addition onto a selection of nitriles are shown in Figure S8. We find that addition onto glyconitrile is indeed favored (by ∼2.5 kcal/mol) compared to HCN dimerization. Similarly, cyanamide, methyliminoacetonitrile, methylcyanamide, and glyconitrile are found to markedly lower the initial reaction step (ΔΔ*E*^TS^ of −4.6 to −3.1 kcal/mol). We also note that cyanide addition onto a protonated form of **2** is barrierless. Low-temperature HCN-based chemical diversification is therefore not only expected but is explainable provided a feasible model for reaction initiation is identified for a given environment.

## Conclusions

The exceedingly high polarity, reactive nature, and abundance of HCN in a host of environments support its role as a source of largely unknown complex chemistry in the solar system and beyond.^12,18^ In this work, we use simulations to corroborate experimental evidence from various HCN polymerization experiments. We demonstrate that both DAMN (**4**) and polyimine (**7**) are viable products of base-catalyzed reactions of HCN with itself. Whereas **4** is the thermodynamic product, kinetic control (cold conditions) can facilitate the formation of oligomers or polymers of **7**. The predicted occurrence, relatively high energy, and photochemical and catalytical properties of **7**(18) support a premise in which it, and structures related to it, can help drive the formation of more complex structures of astrochemical, prebiotic, or astrobiological relevance.

The investigated routes to **4** and **7** are both rate-limited by the base-catalyzed dimerization of HCN, established to proceed rapidly in ambient conditions.^44^ If HCN dimerization (the initial step) can be circumvented, then subsequent chemical reactions can proceed at temperatures as low as ∼150 K. Alternative forms of initiation of these reactions, either through chemical, physical, or irradiative stimuli, would therefore help explain suspected HCN-based chemistry in a host of astrochemical environments, including on Titan, in comets, and, in principle, on a range of different classes of exoplanets. A non-exhaustive screening has identified a series of nitriles as plausible initiators in certain conditions. The identification of other modes for initiating reactivity (and polymerization) of HCN is likely to prove important for understanding the origins of prebiotic chemical diversification and is a promising avenue of future research.

The reaction mechanisms investigated here are but a fraction of those responsible for chemical complexity in astro- and prebiotic chemistry settings. However, because they constitute the very beginning of HCN’s self-reactions, with HCN itself being an almost universally ubiquitous building block, they are possibly among the most fundamental reactions for the subsequent formation of chemical complexity, even life.

## Supporting Information Available

Energies and structures of the conformational search; link to the database with molecular structures and simulation inputs; comparison of barriers for continued polymerization of **7** growth computed with an implicit solvation model; parameters used in umbrella sampling simulations and details of reference simulations and transition state equilibrations; figure with histograms from umbrella sampling simulations; figure of the one-dimensional free energy profile and statistical error bars; figure of different nitriles and predicted energy activation barriers for cyanide addition onto them; and detailed description of path collective variables and parameters used to construct them (PDF)

## Abbreviations

HCN: hydrogen cyanide
DFT: density functional theory
PBE: Perdew–Burke–Ernzerhof
WHAM: Weighted Histogram Analysis Method
