# Supplementary material for: Crossroads at the Origin of Prebiotic Chemical Complexity: Hydrogen Cyanide Product Diversification
Source: J Phys Chem A. 2023 May 11;127(20):4503–10. doi: 10.1021/acs.jpca.3c01504 (PMC10226121; doi:10.1021/acs.jpca.3c01504)
Supplement: Supplementary file 1 — jp3c01504_si_001.pdf [file jp3c01504_si_001.pdf]

Supplementary Information for
Crossroads at the Origin of Prebiotic Chemical Complexity: Hydrogen Cyanide Product Diversification

Authors: Hilda Sandström[1] and Martin Rahm[1,].*

[1] Department of Chemistry and Chemical Engineering, Chalmers University of Technology. Gothenburg 412 96 Sweden.
[*]Corresponding author: E-mail: martin.rahm@chalmers.se, Address: Kemigården 4, 412 58 Gothenburg, Sweden.

This PDF file includes:

- Supplementary text
- Figures S1 to S8
- Tables S1 to S10
- SI References

Structures and simulation inputs used in this work are available at the Swedish National Data Service via the following link: https://doi.org/10.5878/4ysr-8544

Conformational Search

The lowest-energy conformations used in our simulations were identified by manual searches over reactants, intermediates, transition states, and products in the chemical pathways 1-3 (Figure S1-S3). Structures and energies were calculated with Gaussian 16 revision B01[1] using the Perdew-Burke-Ernzerhof (PBE) functional[2] combined with Grimme's D3 dispersion correction[3] and a 6-31G(d,p) basis set. This level of theory is nearly identical to that used in subsequent molecular dynamics simulations. Solvation effects were included using a polarizable continuum model (PCM)[4] for water but with the dielectric constant changed to that of HCN, 144.8[5] at 278 K. Relative energies were also evaluated using the hybrid functionals B3LYP-D3[6,7] and M06-2X[8] with a 6-31+G(d,p) basis set for comparison. The M06-2X calculations were done as single point energy calculations on the B3LYP-D3 optimized structures. The thermal corrections at the B3LYP-D3 level of theory were added to compute the free energy. The hybrid functionals predict relative energies of ground state conformers within ~1kcal/mol of PBE-D3 (Figure S1) but is too computationally demanding for molecular dynamics simulations. Transition states identified to be lowest in energy (at all three levels of theory, Figure S3) were used as input for subsequent dynamics simulations that generated pathways from reactants to products.

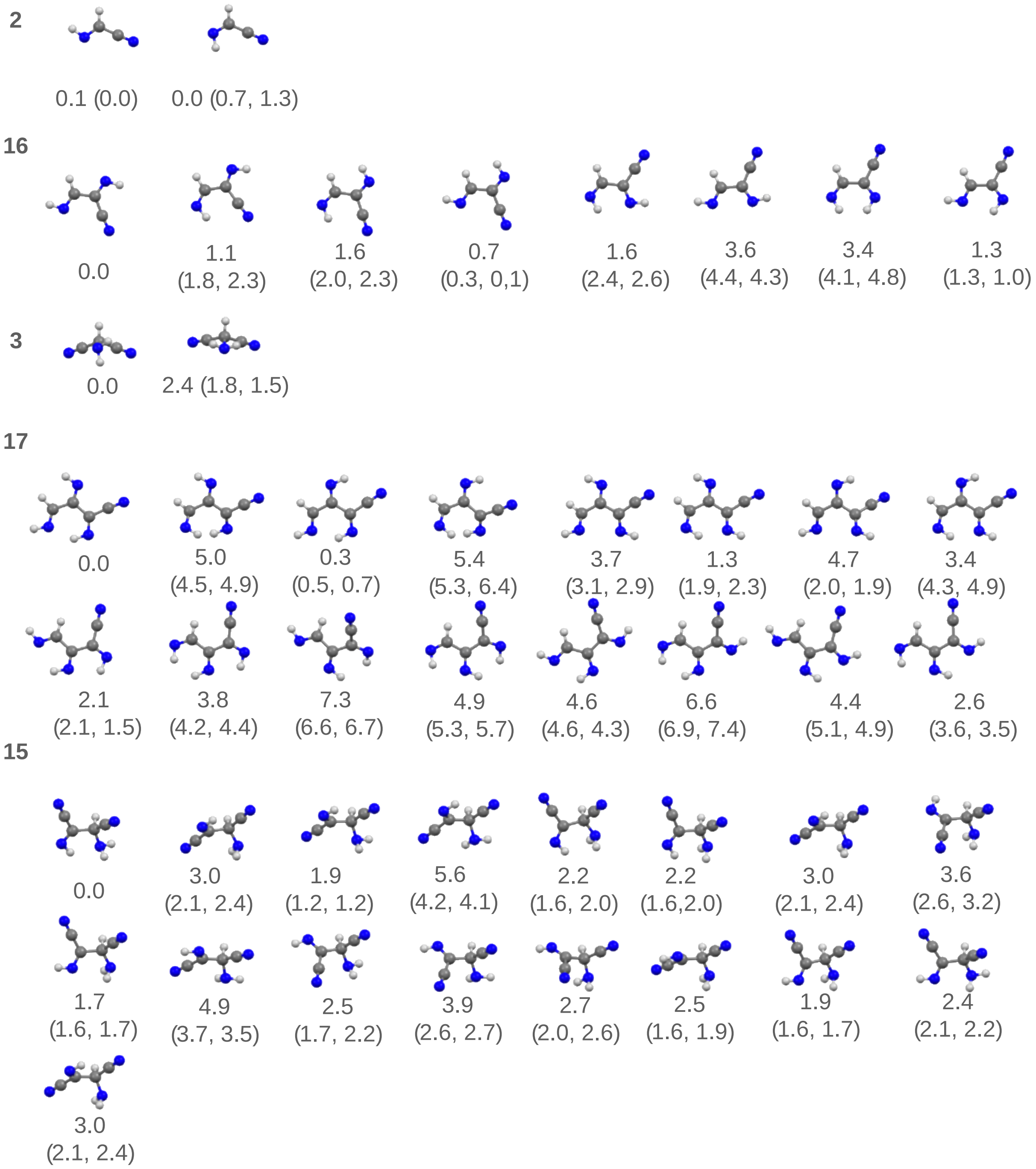

Figure S1. Geometries and relative energies of molecular conformers involved in pathways 1-3. Relative Gibbs free energies (ΔG_{278K}) are computed at the PCM-PBE-D3/6-31G(d,p) level of theory. Corresponding values evaluated at the PCM-B3LYP-D3/6-31+G(d,p) (left) and the PCM-M06-2X-6-31+G(d,p)//PCM-B3LYP-D3/6-31+G(d,p) (right) level of theory are provided within parentheses.

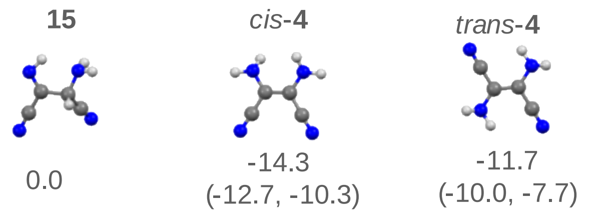

Figure S2. Structure and relative Gibbs free energy at 278 K of the tautomers 2-amino-3-imino butanedinitrile (**15**) and diaminomaleonitrile (**4**) computed at the PCM-PBE-D3/6-31G(d,p) level of theory. Corresponding values evaluated at the PCM-B3LYP-D3/6-31+G(d,p) (left) and the PCM-M06-2X-6-31+G(d,p)//PCM-B3LYP-D3/6-31+G(d,p) (right) level of theory are provided within parentheses.

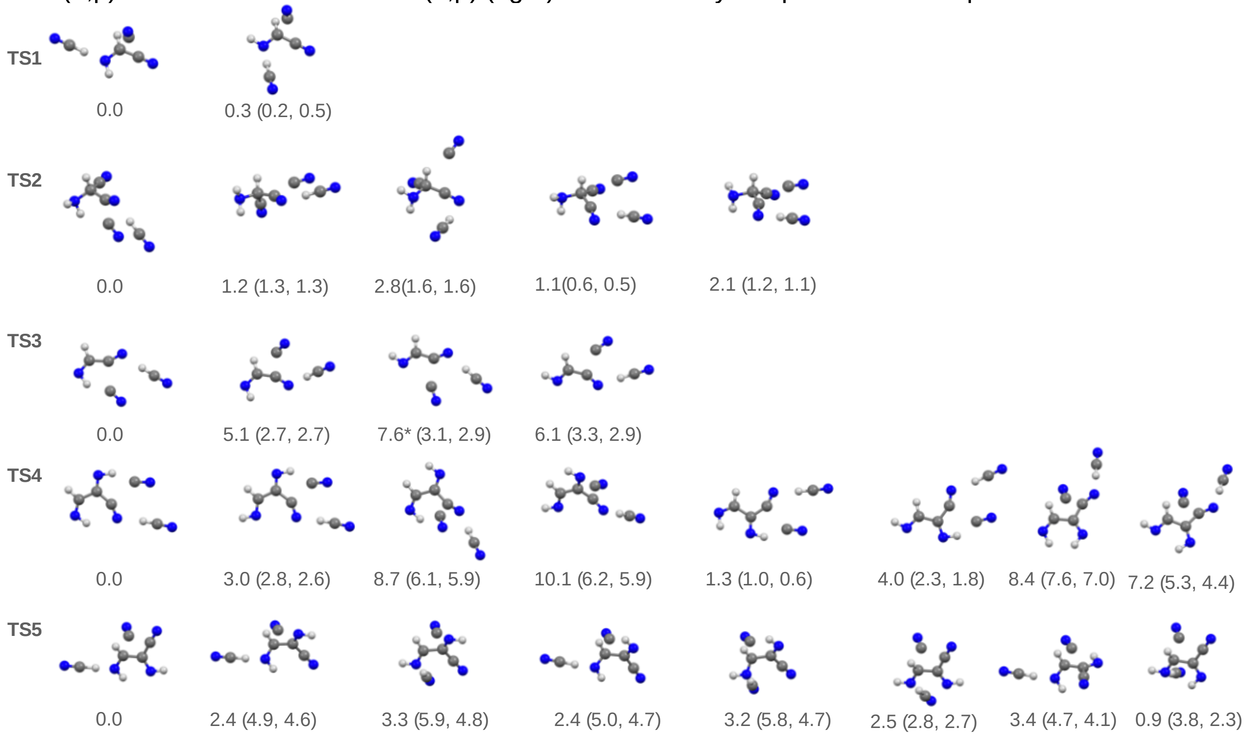

Figure S3. Structures and relative free energies of different transition state conformations in pathways 1-3. Relative Gibbs free energies (ΔG_{278K}) have been computed at the PCM-PBE-D3/6-31G(d,p) level of theory. Corresponding values at the PCM-B3LYP-D3/6-31+G(d,p) (left) and the PCM-M06-2X-6-31+G(d,p)//PCM-B3LYP-D3/6-31+G(d,p) (right) level of theory are provided within parentheses. *The electronic energy contribution was computed for a structure optimized at the PCM-B3LYP-D3/6-31+G(d,p) level of theory.

Convergence of Barrier to Polymerization into Polyimine, as a Function of Chain Length

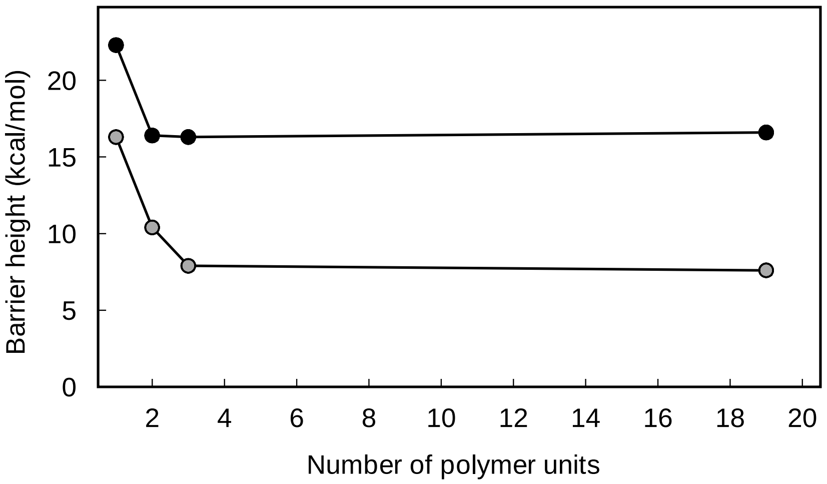

Figure S4. Gibbs free energy barriers (278 K) for the early stages of polyimine formation as a function of chain length. Grey and black circles correspond to PCM-PBE-D3/6-31G(d,p) and PCM-B3LYP-D3/6-31+G(d,p) levels of theory, respectively. The implicit PCM solvent model is that of water but with an adjusted dielectric constant of 144.8.

Construction of path collective variables for steered simulations.
Umbrella sampling simulations were performed along reaction paths defined by path collective variables.[9] The path collective variables *s*, and *z* are defined as:

$$s(t) = \frac{\sum_{k=1}^{N_k} k e^{-\lambda D_k(t)}}{\sum_{k'=1}^{N_k} e^{-\lambda D_k(t)}}$$

$$z(t) = -\frac{1}{\lambda} log\left(\sum_{k=1}^{N_k} e^{-\lambda D_k(t)}\right),$$

where N_k refers to the number of reference structures and λ is a parameter determining the distance between the reference structures in *s*,*z* space. D_k is a distance function. For this study we made use of the coordination-based distance function by Pietrucci and Saitta,[10] $D_k(t) = \sum_{IS}[C_{IS}(t) - C_{IS}^k]^2$, where $C_{IS}(t)$ is the coordination between atom I and atoms of type S and C_{IS}^k is the corresponding coordination in reference structure *k*. The coordination patters are calculated as,

$$C_{IS}(t) = \sum_{J \in S} \frac{\left[1-\left(\frac{R_{IJ}(t)}{R_{ss'}^0}\right)^N\right]}{\left[1-\left(\frac{R_{IJ}(t)}{R_{ss'}^0}\right)^M\right]} ,$$

where N and M are set to 8 and 16 following the recommendations of Pérez-Villa et al.,[11] and $R_{IJ}(t)$ corresponds to the Euclidian distance between atoms I and J. $R_{ss'}^0$ is a reference distance which was set to 1.8 Å for distances between non-hydrogen atoms and 1.5 Å for distances between hydrogen atoms and non-hydrogen atoms. In this work, we used two separate reference structures for each studied reaction, corresponding to reactants and products. The λ-parameter was adjusted so that $\lambda = \frac{2.3}{D_k(t)}$.[11]

We initially ran a reference simulation of **2** performed with a cutoff of 200 Ry and a Nose-Hoover thermostat of length 4 and a time constant of 500 fs.[12] In all subsequent reference states, we used the canonical sampling through velocity rescaling thermostat[13] for both equilibration and production runs (with a time constant of 50 fs for production runs and 1 fs for equilibrations) since it worked better with our steered simulations. We used a more accurate cutoff of 280 Ry for all other simulations besides those of **2**. We nonetheless still utilized the reference state simulation of **2** for analysis. We do not expect the cutoff and thermostat to change the atomic coordination of the atoms in the reference simulations in a meaningful way. However, we note that convergence with a cutoff of 280 Ry is better (< 1 meV/atom) than with 200 Ry (< 2 meV/atom). 280 Ry has also been shown to produce accurate forces and energies for NVT ensembles of water.[14]

Tables S1-S5 shows the coordination patterns used to construct the *s* and *z* variables for all reaction steps in pathways 1-3. For each step, we defined coordination patterns of the atoms of the cyanide anion. We also included the coordination of the carbon subjected to the nucleophilic attack by the cyanide anion as well as that of the nitrogen bonded to it. The coordination in the reference states was calculated based on the average distance between atoms during 20 ps production runs preceded with a 5 ps equilibration.

In the reference state simulations of **2** and **3** there occurred a proton transfer to the cyanide anion. Analysis of the C-H coordination based on the whole production run of the cyanide anion gave C_{CH}^k of 0.69 and 0.73 for the simulation of **2** and **3** respectively. This coordination pattern was used during the transition state equilibration. For subsequent committor analysis and umbrella sampling simulations a coordination pattern based on the section of the production run prior to the proton transfer was used. Prior to the proton transfer C_{CH}^k was equal to 0.21 in the simulations of **2** and **3** both.

Table S1. Coordination patterns, C_{IS}^{k}, for the first reaction step of pathway 1. The coordination patterns are for atoms in the cyanide anion (denoted by subscript a), the *sp*^{2} carbon of iminoacetonitrile (**2**) and the nitrogen of the imino-group (reactant state). The corresponding values are also shown for the same atoms in the product aminomalononitrile (**3**). A value close to 1 indicates a covalent bond.

Atom group S / Atom I	C	N	H
Reactant			
C	0.85	1.00	1.04
N	1.04	0.03	1.07
C_a	0.03	0.99	0.21
N_a	1.00	0.01	0.13
Product			
C	1.64	0.94	1.07
N	1.00	0.04	2.02
C_a	0.91	1.07	0.08
N_a	1.04	0.02	0.04

Table S2. Coordination patterns, C_{IS}^{k}, used for the second reaction step in pathway 1 (from aminomalononitrile (**3**) to 2-amino-3-imino butanedinitrile (**15**)). The coordination pattern of the atoms in the cyanide anion (denoted by subscript a) and a nitrile group of **3** were used in the reactant state. The coordination patterns of the same atoms in the product **15** are also shown. A value close to 1 indicates a covalent bond.

Atom group S / Atom I	C	N	H
Reactant			
C	0.91	1.07	0.08
N	1.04	0.02	0.04
C_a	0.03	0.99	0.21
N_a	1.00	0.02	0.13
Product			
C	1.69	1.07	0.19
N	1.13	0.06	1.00
C_a	0.92	1.07	0.03
N_a	1.04	0.02	0.03

Table S3. Coordination patterns, C_{IS}^{k}, of atoms in the first reaction step of pathway 2. The coordination patterns are reported for the atoms in the cyanide anion (denoted by subscript a) and the nitrile group of iminoacetonitrile (**2**) (reactant state). The corresponding values for the same atoms in product diiminopropanenitrile (**16**) are also shown. A value close to 1 indicates a covalent bond.

Atom group S / Atom I	C	N	H
Reactant			
C	0.85	1.07	0.07
N	1.03	0.02	0.06
C_a	0.03	0.99	0.21
N_a	1.00	0.01	0.13
Product			
C	1.64	1.06	0.18
N	1.12	0.03	1.00
C_a	0.91	1.10	0.03
N_a	1.04	0.03	0.04

Table S4. Coordination patterns, C_{IS}^{k}, of atoms in the second reaction step of pathway 2. The coordination of atoms in the cyanide anion (denoted by subscript a), and the nitrile group of diiminopropanenitrile (**16**) state were used for the reactant state. The corresponding values of the atoms are also shown in the product triiminobutanenitrile (**17**). A coordination value close to 1 indicates a covalent bond.

Atom group S / Atom I	C	N	H
Reactant			
C	0.91	1.10	0.03
N	1.04	0.03	0.04
C_a	0.04	0.97	0.19
N_a	1.01	0.01	0.25
Product			
C	1.70	1.09	0.13
N	1.14	0.04	0.98
C_a	0.91	1.09	0.02
N_a	1.04	0.03	0.03

Table S5. Coordination patterns, C_{IS}^{k}, of atoms in the second step of pathway 3. For the reactant state, the coordination of atoms in the cyanide anion (denoted by subscript a), a sp^2 carbon of diiminopropanenitrile (**16**), and the nitrogen bonded to that carbon are shown. The coordination of the same atoms in the product 2-amino-3-imino butanedinitrile (**15**) are displayed below. Values close to 1 indicates a covalent bond.

Atom group S / Atom I	C	N	H
Reactant	C	N	H
C	0.87	1.03	1.05
N	1.05	0.03	1.09
C_a	0.04	0.97	0.19
N_a	1.01	0.01	0.25
Product			
C	1.65	0.98	1.07
N	1.01	0.07	2.03
C_a	0.92	1.06	0.09
N_a	1.04	0.02	0.06

Force Constants in transition state equilibrations and umbrella sampling simulations.

The force constants were selected to not exceed $\frac{k_B T}{\sigma^2}$, where k_B is the Boltzmann constant, T refers to the temperature (278 k) and σ is the standard deviation of the s coordinate in a typical simulation of the reactant or product state.

The input transition state (TS) guess structures were DFT optimized as minimal molecular models (later solvated in HCN prior to simulation). **TS1-3** were optimized in gas phase with a 6-311++G(d,p) basis set using the B3LYP functional and Grimme D3 corrections. Transition states **TS4** and **TS5** were optimized with PBE-D3 and a 6-31G(d,p) basis set. In all cases the TSs were allowed to equilibrate for 20-25 ps. During TS equilibration, the s value of all TSs was restrained with a force constant of 10 000 kJ/mol. We added an additional weak bias of 3000 kJ/mol to the z variable during the equilibration of **TS5.** The z bias was introduced to hinder an otherwise observed immediate proton transfer to **16** at the start of the equilibration. A protonated **16** computes as 20 kcal/mol above the corresponding neutral molecule at the PCM-PBE-D3/6-31G(d,p) level of theory**.** Therefore, the spontaneous formation of a protonated **16** was likely due to the coarse initial solvent configuration prior to equilibration.

Input structures for the umbrella sampling windows were taken from trajectories reaching between the equilibrated TS to the reactant and product basins. The umbrella sampling windows were constructed so that they were at most 0.033 s units apart, corresponding to approximately 4 % of the distance between the reactants and products for all studied reaction steps. The s value in each umbrella sampling window was constrained using a harmonic potential on the form $k\ (s(t) - s_0)^2$. In Tables S6-S10 we tabulate the force constant, k, and s value associated with each umbrella sampling window, s_0.

Table S6. s_0 and k parameters used to describe the harmonic potentials along the reaction coordinate between 2,3-diiminopropanenitrile (**16**) and iminoacetonitrile (**2**). s_0 denotes the center of the potential and k is the force constant of the potential.

s_0	k [kJ/mol]	s_0	k [kJ/mol]
1.099055	9000	1.541712	9000
1.114885	9000	1.56708	9000
1.140864	9000	1.590553	9000
1.165709	9000	1.61566	9000
1.190247	9000	1.638557	9000
1.215514	9000	1.661539	9000
1.220000	12000	1.690304	9000
1.240262	9000	1.715713	9000
1.264373	9000	1.738821	9000
1.290594	9000	1.76412	9000
1.316726	9000	1.792657	12000
1.340355	9000	1.792657	9000
1.366360	9000	1.803002	13000
1.390862	9000	1.815315	12000
1.411378	9000	1.815315	9000
1.443877	9000	1.835941	9000
1.465462	9000	1.868229	9000
1.493829	9000	1.891712	9000
1.516240	9000	1.909519	9000

Table S7. s_0 and k parameters used to describe the harmonic potentials along the reaction coordinate between aminomalononitrile (**3**) and iminoacetonitrile (**2**). s_0 denotes the center of the potential and k is the force constant of the potential.

s_0	k [kJ/mol]	s_0	k [kJ/mol]
1.09861	10000	1.515103	10000
1.115079	10000	1.544444	10000
1.140812	10000	1.558883	10000
1.164734	10000	1.590557	10000
1.194206	10000	1.620826	10000
1.216784	10000	1.645467	10000
1.241396	10000	1.665986	10000
1.264654	10000	1.691689	10000
1.290043	10000	1.716245	10000
1.318173	10000	1.741642	10000
1.339858	10000	1.767181	10000
1.365765	10000	1.790163	10000
1.39017	10000	1.813886	10000
1.412844	10000	1.840935	10000
1.441747	10000	1.864024	10000
1.468682	10000	1.890562	10000
1.491641	10000	1.901316	10000

Table S8. s_0 and k parameters used to describe the harmonic potentials along the reaction coordinate between 2,3-diiminopropanenitrile (**16**) and 2,3,4-triiminobutanenitrile (**17**). s_0 denotes the center of the potential and k is the force constant of the potential.

s_0	k [kJ/mol]	s_0	k [kJ/mol]
1.084218	10000	1.541816	10000
1.090643	10000	1.568628	10000
1.110281	10000	1.598206	10000
1.140573	10000	1.613256	10000
1.164895	10000	1.643185	10000
1.188927	10000	1.657422	10000
1.188927	13000	1.68554	10000
1.216692	10000	1.713975	10000
1.241208	10000	1.741967	10000
1.264404	10000	1.768526	10000
1.290513	10000	1.795831	10000
1.311043	10000	1.795831	15000
1.340548	10000	1.82161	10000
1.368036	10000	1.82161	13000
1.39284	10000	1.82161	15000
1.418306	10000	1.844882	10000
1.442235	10000	1.864923	10000
1.466841	10000	1.894144	10000
1.495133	10000	1.915905	10000
1.511645	10000		

Table S9. s_0 and k parameters used to describe the harmonic potentials along the reaction coordinate between 2,3-diiminopropanenitrile (**16**) and 2-amino-3-imino butanedinitrile (**15**). s_0 denotes the center of the potential and k is the force constant of the potential.

s_0	k [kJ/mol]	s_0	k [kJ/mol]
1.091802	10000	1.513054	10000
1.115089	10000	1.540854	10000
1.140519	10000	1.562933	10000
1.164874	10000	1.596041	10000
1.190631	10000	1.610727	10000
1.2149	10000	1.644141	10000
1.239146	10000	1.644141	13000
1.266798	10000	1.664777	10000
1.266798	13000	1.68555	10000
1.294362	10000	1.717686	10000
1.31927	10000	1.741049	10000
1.342334	10000	1.768787	10000
1.342334	13000	1.796034	10000
1.367479	10000	1.813626	10000
1.386288	10000	1.839647	10000
1.417459	10000	1.864897	10000
1.437643	10000	1.893787	10000
1.465283	10000	1.908909	10000
1.490687	10000	1.918661	10000

Table S10. s_0 and k parameters used to describe the harmonic potentials along the reaction coordinate between aminomalononitrile (**3**) and 2-amino-3-imino butanedinitrile (**15**). s_0 denotes the center of the potential and k is the force constant of the potential.

s_0	k [kJ/mol]	s_0	k [kJ/mol]
1.097113	10000	1.520144	10000
1.115221	10000	1.542972	10000
1.140917	10000	1.567324	10000
1.165488	10000	1.593561	10000
1.190976	10000	1.612497	10000
1.207098	13000	1.642467	10000
1.2122	10000	1.669433	10000
1.2122	12000	1.701353	10000
1.2122	13000	1.717046	10000
1.242002	10000	1.739541	10000
1.265688	10000	1.764065	10000
1.292857	10000	1.790647	10000
1.313038	10000	1.790647	13000
1.339997	10000	1.817036	10000
1.362704	10000	1.817036	13000
1.394323	10000	1.840127	10000
1.417812	10000	1.865975	10000
1.443336	10000	1.890447	10000
1.468113	10000	1.896438	10000
1.494145	10000		

Histograms from Umbrella Sampling Simulations

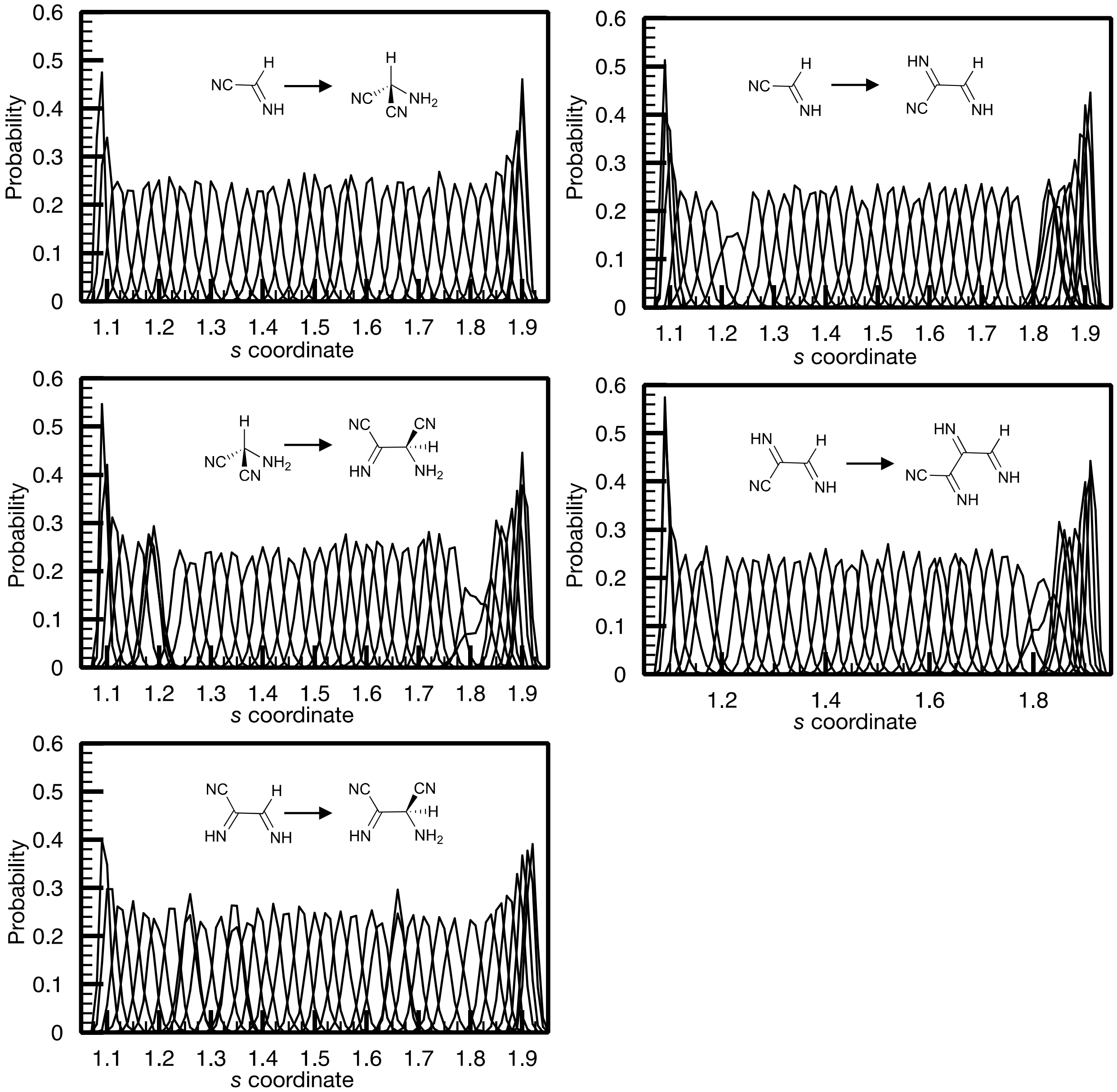

Figure S5. Histograms of sampled reaction coordinates in the umbrella sampling windows for all the reaction steps in pathways 1, 2 and 3. A good overlap between adjacent histogram is necessary for a reliable reconstruction of the relative Gibbs free energy.

Free Energy Curves from Umbrella Sampling Simulations

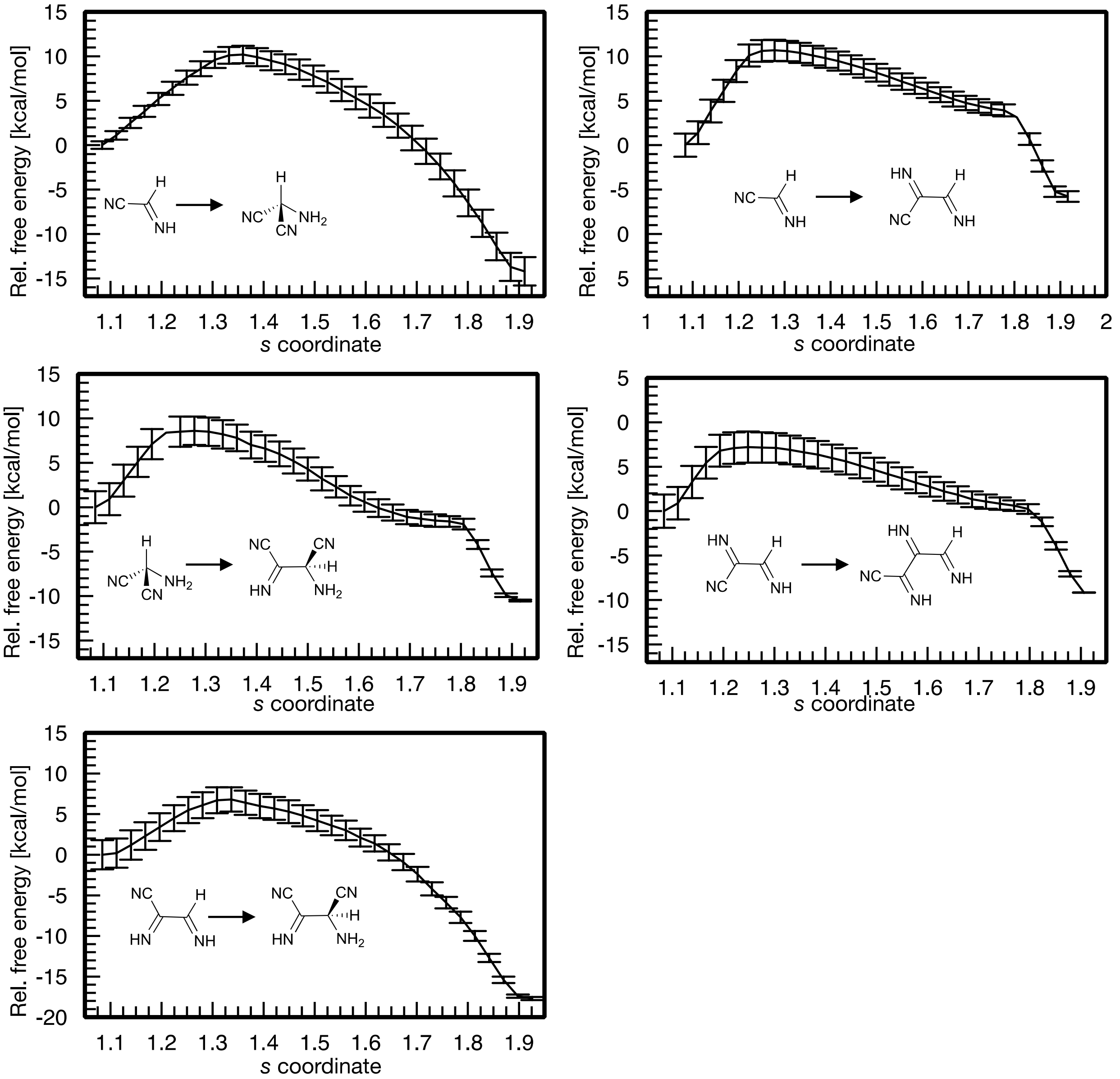

Figure S6. The free energy profiles for all reaction steps in pathways 1-3, obtained using umbrella sampling at the PBE-D3/DZVP-GTH level of theory. Error bars correspond to standard deviations that were obtained using block averaging as implemented in the weighted histogram analysis method (WHAM) program.[15]

Statistical Error of Energy Profiles

The uncertainty of the relative energy between two points on a free energy curve depends on the uncertainty of the energy in both points. Therefore, we can use the standard deviations of the umbrella sampling curves (Fig. S7) to estimate the uncertainty of the relative energy of the transition state or product compared to the reactants. Under the assumption that the energies are independent and follow a Gaussian distribution, the error $\sigma(\Delta E_i(j))$ of the relative energy of products and transition states can be computed according to $\sigma(\Delta E_i(j)) = \sqrt{var\ (E_i(j)) + var\ (E_i(reactant))}$, where i= 3 or 4 (corresponding to a trimerization (3) or tetramerization (4), respectively) and j refers to the transition state or product.

The relative energy error of the trimerization profile, ΔE_3, will influence the overall error of the relative energy of the tetramer compared to the dimer. To construct a complete energy profile ΔE_F that connects **2** to the tetramers, the trimerization and tetramerization energy profiles (ΔE_3 and ΔE_4 respectively) can be added according to $\Delta E_F = \Delta E_3 + \Delta E_4$. The standard deviation of final relative energy of the states along the tetramerization energy profile are then computed as $\sigma = \sqrt{var\ (\Delta E_3(trimer)) + var\ (\Delta E_4(i))}$, where i is either a tetramer (**15** or **17**) or a second transition state of the pathway (either **TS2**, **TS4**, **TS5**). The final standard deviations of relative energies along the pathways lie between 1.1 and 2.9 kcal/mol (Fig. S8).

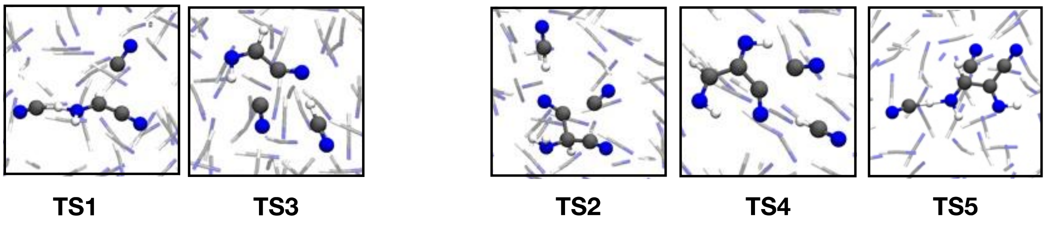

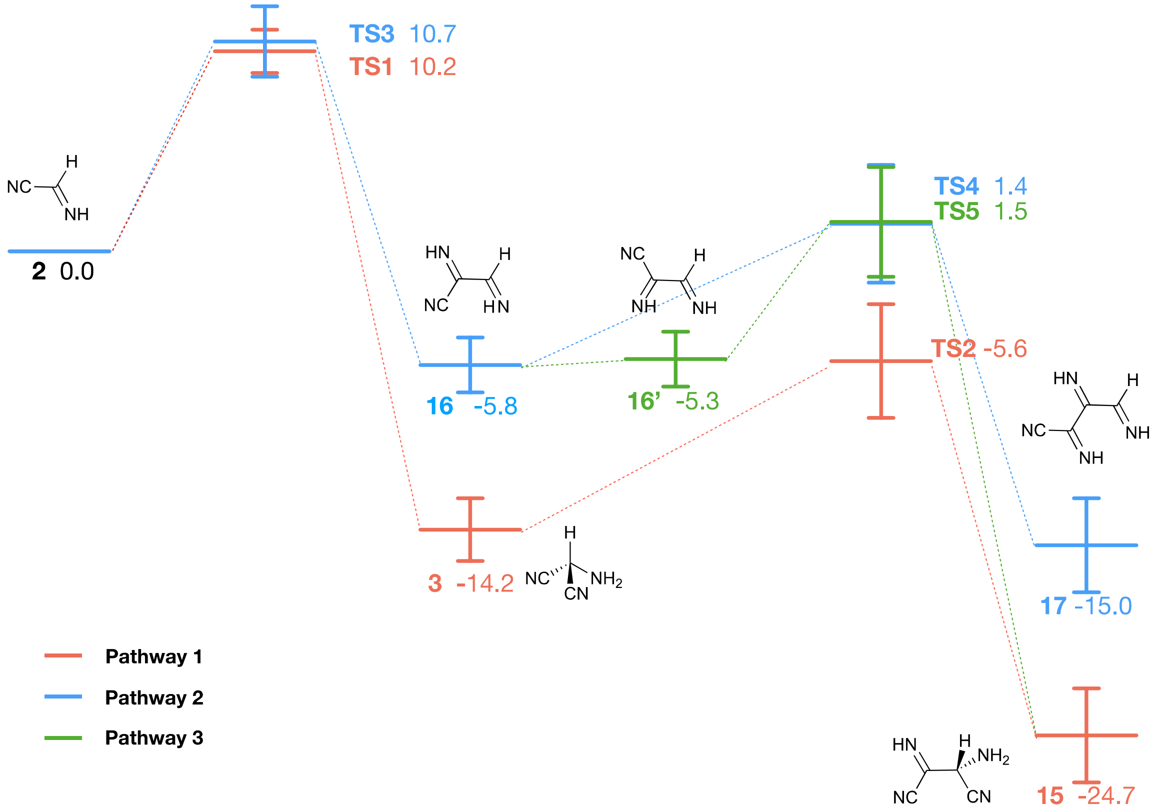

Figure S7. The Gibbs free energy for formation of 2-amino-3-imino butanedinitrile (**15**) and 2,3,4-triiminobutanenitrile (**17**) at 278 K as presented in Figure 3 of the main text, here shown together with the associated error bars. The errors correspond to standard deviation of the relative energy and were computed as described in the text above.

Cyanide Addition on Other Nitriles

The first dimerization step in Pathways 1-3 is rate-limiting and consists of a cyanide addition onto HCN. We have explored whether other nitriles could act as initiators for circumventing this first step. To accelerate reactivity, cyanide additions onto these nitriles would need to have lower barriers than the addition onto HCN. Possible initiators were selected following two criteria; they should be 1) small and 2) contain different functional groups attached to the -CN group. Figure 8 shows barrier heights for CN- addition relative to HCN ($\Delta\Delta E^{TS}$). Only the electronic (Born-Oppenheimer) energy contribution is considered here, and we assume enthalpic and entropic effects to be relatively minor. The reference states for these barrier heights are free reactants. Calculations were made using Gaussian 16's default PCM model for water at the B3LYP-D3/6-311++G(d,p) level of theory.

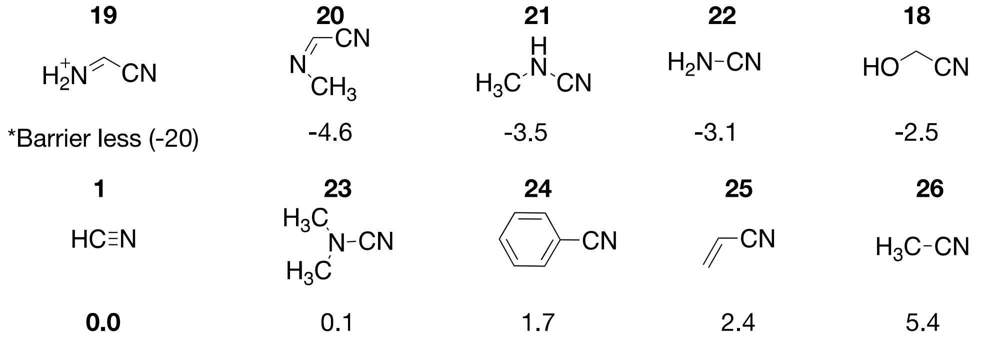

Figure S8. Activation energy barrier height for cyanide addition with a selection of small nitriles, shown relative to the reaction with HCN ($\Delta\Delta E^{TS}$, kcal/mol).

Full Reference to Gaussian16

Frisch, M. J.; Trucks, G. W.; Schlegel, H. B.; Scuseria, G. E.; Robb, M. A.; Cheeseman, J. R.; Scalmani, G.; Barone, V.; Petersson, G. A.; Nakatsuji, H.; Li, X.; Caricato, M.; Marenich, A. V.; Bloino, J.; Janesko, B. G.; Gomperts, R.; Mennucci, B.; Hratchian, H. P.; Ortiz, J. V.; Izmaylov, A. F.; Sonnenberg, J. L.; Williams-Young, D.; Ding, F.; Lipparini, F.; Egidi, F.; Goings, J.; Peng, B.; Petrone, A.; Henderson, T.; Ranasinghe, D.; Zakrzewski, V. G.; Gao, J.; Rega, N.; Zheng, G.; Liang, W.; Hada, M.; Ehara, M.; Toyota, K.; Fukuda, R.; Hasegawa, J.; Ishida, M.; Nakajima, T.; Honda, Y.; Kitao, O.; Nakai, H.; Vreven, T.; Throssell, K.; Montgomery, J. A., Jr.; Peralta, J. E.; Ogliaro, F.; Bearpark, M. J.; Heyd, J. J.; Brothers, E. N.; Kudin, K. N.; Staroverov, V. N.; Keith, T. A.; Kobayashi, R.; Normand, J.; Raghavachari, K.; Rendell, A. P.; Burant, J. C.; Iyengar, S. S.; Tomasi, J.; Cossi, M.; Millam, J. M.; Klene, M.; Adamo, C.; Cammi, R.; Ochterski, J. W.; Martin, R. L.; Morokuma, K.; Farkas, O.; Foresman, J. B.; Fox, D. J. Gaussian 16, Revision B.01. Gaussian, Inc., Wallingford CT; 2016

SI References

1. Frisch, M. J. et al. *Gaussian 16, Revision B.01* (Gaussian, Inc., Wallingford CT, 2016).
2. Perdew, J. P.; Burke, K.; Ernzerhof, M. Generalized Gradient Approximation Made Simple. *Phys. Rev. Lett.* **1996**, *77*, 3865-3868.
3. Grimme, S.; Ehrlich, S.; Goerigk, L. Effect of the Damping Function in Dispersion Corrected Density Functional Theory. *J. Comput. Chem.* **2011**, *32*, 1456-1465.
4. Tomasi, J.; Mennucci, B.; Cammi, R. Quantum Mechanical Continuum Solvation Models. *Chem. Rev.* **2005**, *105*, 2999-3093.
5. Coates, G. E.; Coates, J. E. Hydrogen Cyanide. Part Xiii. The Dielectric Constant of Anhydrous Hydrogen Cyanide. *J Chem Soc* **1944**, 77-81.
6. Becke, A. D. A New Mixing of Hartree-Fock and Local Density-Functional Theories. *J. Chem. Phys.* **1993**, *98*, 1372-1377.
7. Stephens, P. J.; Devlin, F. J.; Chabalowski, C. F.; Frisch, M. J. Ab Initio Calculation of Vibrational Absorption and Circular Dichroism Spectra Using Density Functional Force Fields. *J. Phys. Chem.* **1994**, *98*, 11623-11627.
8. Zhao, Y.; Truhlar, D. G. The M06 Suite of Density Functionals for Main Group Thermochemistry, Thermochemical Kinetics, Noncovalent Interactions, Excited States, and Transition Elements: Two New Functionals and Systematic Testing of Four M06-Class Functionals and 12 Other Functionals. *Theor. Chem. Acc.* **2008**, *120*, 215-241.
9. Branduardi, D.; Gervasio, F. L.; Parrinello, M. From a to B in Free Energy Space. *J. Chem. Phys.* **2007**, *126*, 054103.
10. Pietrucci, F.; Saitta, A. M. Formamide Reaction Network in Gas Phase and Solution Via a Unified Theoretical Approach: Toward a Reconciliation of Different Prebiotic Scenarios. *Proc. Natl. Acad. Sci. U. S. A.* **2015**, *112*, 15030.
11. Pérez-Villa, A.; Saitta, A. M.; Georgelin, T.; Lambert, J.-F.; Guyot, F.; Maurel, M.-C.; Pietrucci, F. Synthesis of RNA Nucleotides in Plausible Prebiotic Conditions From Ab Initio Computer Simulations. *J. Phys. Chem. Lett.* **2018**, *9*, 4981-4987.
12. Evans, D. J.; Holian, B. L. The Nose–Hoover Thermostat. *J. Chem. Phys.* **1985**, *83*, 4069-4074.
13. Bussi, G.; Donadio, D.; Parrinello, M. Canonical Sampling Through Velocity Rescaling. *J. Chem. Phys.* **2007**, *126*, 014101.
14. McGrath, M. J.; Siepmann, J. I.; Kuo, I.-F. W.; Mundy, C. J.; VandeVondele, J.; Hutter, J.; Mohamed, F.; Krack, M. Isobaric–Isothermal Monte Carlo Simulations From First Principles: Application to Liquid Water At Ambient Conditions. *ChemPhysChem* **2005**, *6*, 1894-1901.
15. Grossfield, A. *WHAM: The weighted histogram analysis method*. version 2.0.9. http://membrane.urmc.rochester.edu/content/wham
